# Supplementary material for: Crystal structures of the ATP-binding and ADP-release dwells of the V1 rotary motor
Source: Nat Commun. 2016 Oct 27;7:13235. doi: 10.1038/ncomms13235 (PMC5095293; doi:10.1038/ncomms13235)
Supplement: Supplementary Information — Supplementary Figures 1-14, Supplementary Tables 1-2. [file ncomms13235-s1.pdf]

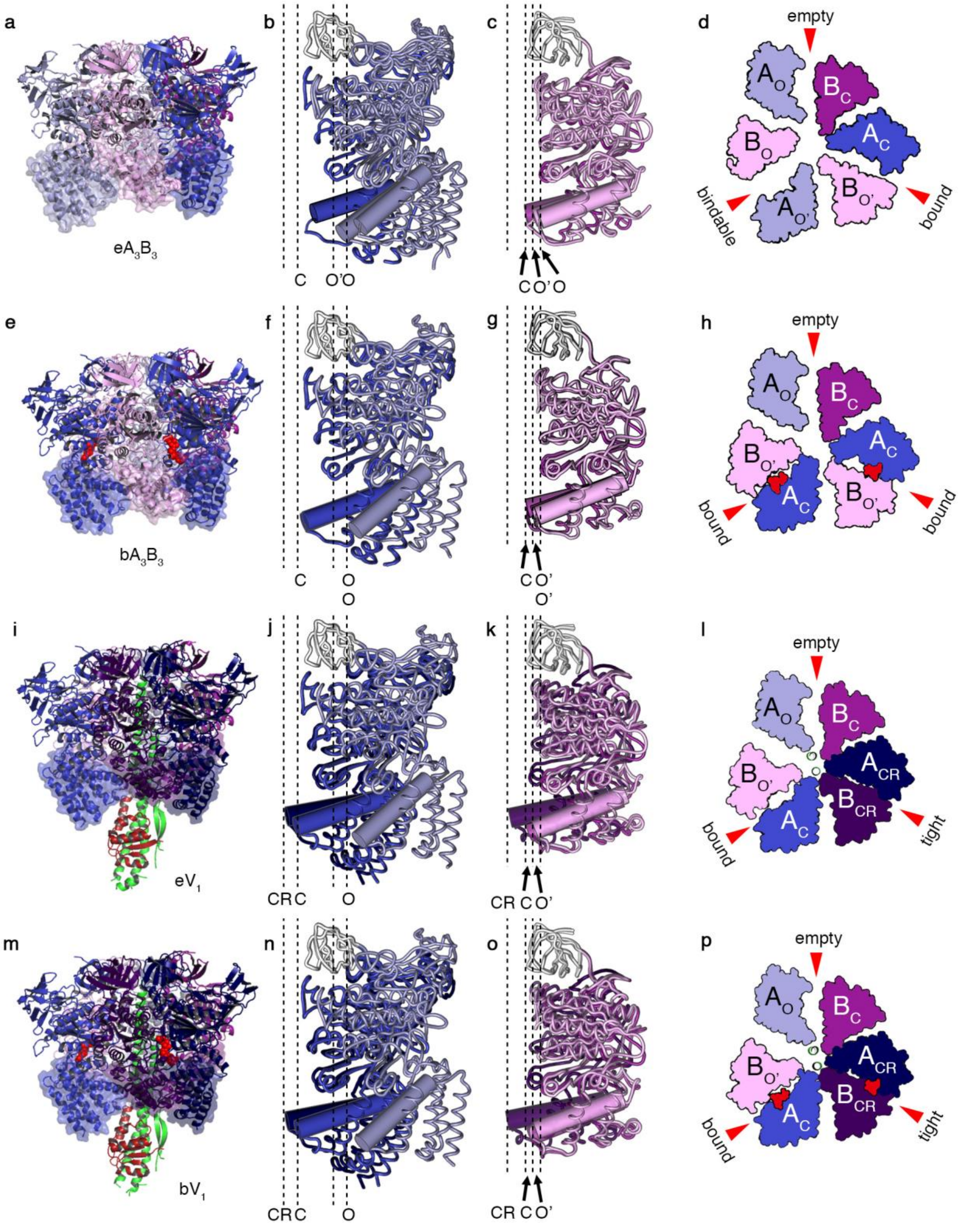

Supplementary Figure 1

Crystal structures of the A_3B_3 and A_3B_3DF (V_1) complexes.

(**a**) Side view of the nucleotide-free A_3B_3 structure (eA_3B_3). Eh-A and Eh-B are shown as blue and purple cartoon representations with a transparent C-terminal domain surface, respectively. (**b**,**c**) Superimposed structures at the N-terminal β-barrel (white) of the 3 structures of Eh-A (**b**) and Eh-B (**c**) in eA_3B_3. Open (O and O') and closed (C) conformations of Eh-A and Eh-B are shown in light and dark colours, respectively. The longest α-helices of the C-terminal domains in Eh-A and -B are shown as cylinders to clarify the structural differences. (**d**) Top view of the C-terminal domain (shown in **a** as a surface) of eA_3B_3 from the cytoplasmic side. Red arrows indicate the nucleotide-binding sites. (**e**–**h**) Structures of the nucleotide-bound A_3B_3 complex (bA_3B_3) viewed and coloured as in **a**–**d** for comparison to the eA_3B_3 structures. (**i**–**l**) Structures of nucleotide-free V_1 (eV_1), viewed and coloured as in **a**–**d**. Open (O and O'; light), closed (C;

dark), and closer (CR; darker) conformations of Eh-A and Eh-B are shown. (**m**–**p**) Structures of nucleotide-bound V_1 (bV_1), viewed and coloured as in **a**–**d**. The bound AMP-PNP molecules are shown in space-filling representation and coloured red.

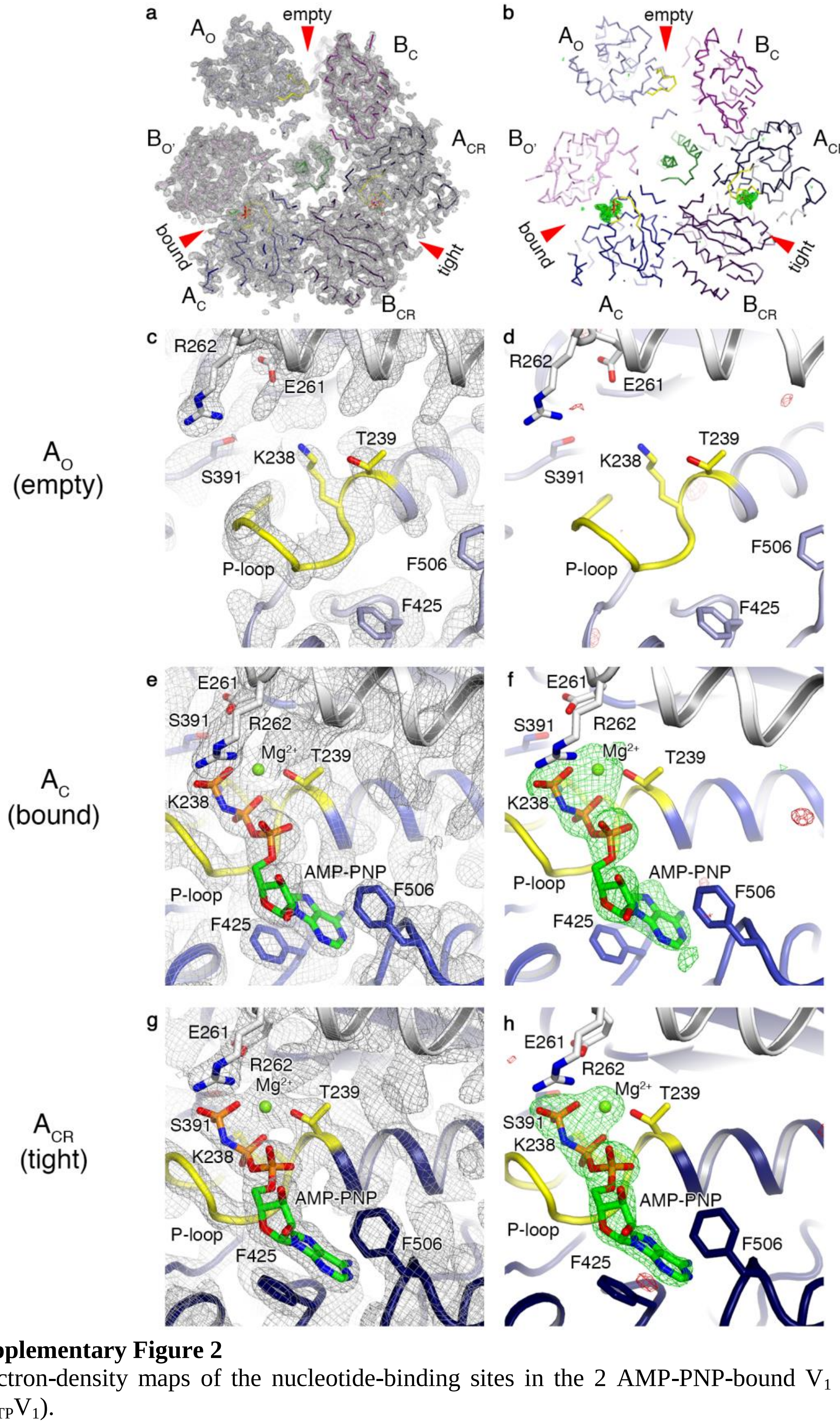

Supplementary Figure 2

Electron-density maps of the nucleotide-binding sites in the 2 AMP-PNP-bound V_1 complex ($2_{ATP}V_1$).

(**a**,**b**) Top views of $2_{ATP}V_1$ from the cytoplasmic side at the level of the nucleotide-binding sites. (**c**–**h**) Nucleotide-binding sites of A_O (**c**,**d**), A_C (**e**,**f**), and A_{CR} (**g**,**h**) of $2_{ATP}V_1$ viewed as in the left panel of Figure 4**a**. Residues involved in nucleotide binding are shown in a stick representation. Colours correspond to those described in Figure 4. Left panels (**a**,**c**,**e**,**g**): the 2|Fo|-|Fc| maps contoured at 1.0 sigma are shown in grey. Right panels (**b**,**d**,**f**,**h**): the |Fo|-|Fc| maps calculated without AMP-PNP:Mg^{2+} at the binding pockets contoured at 4.0 sigma are shown in red (negative) and green (positive).

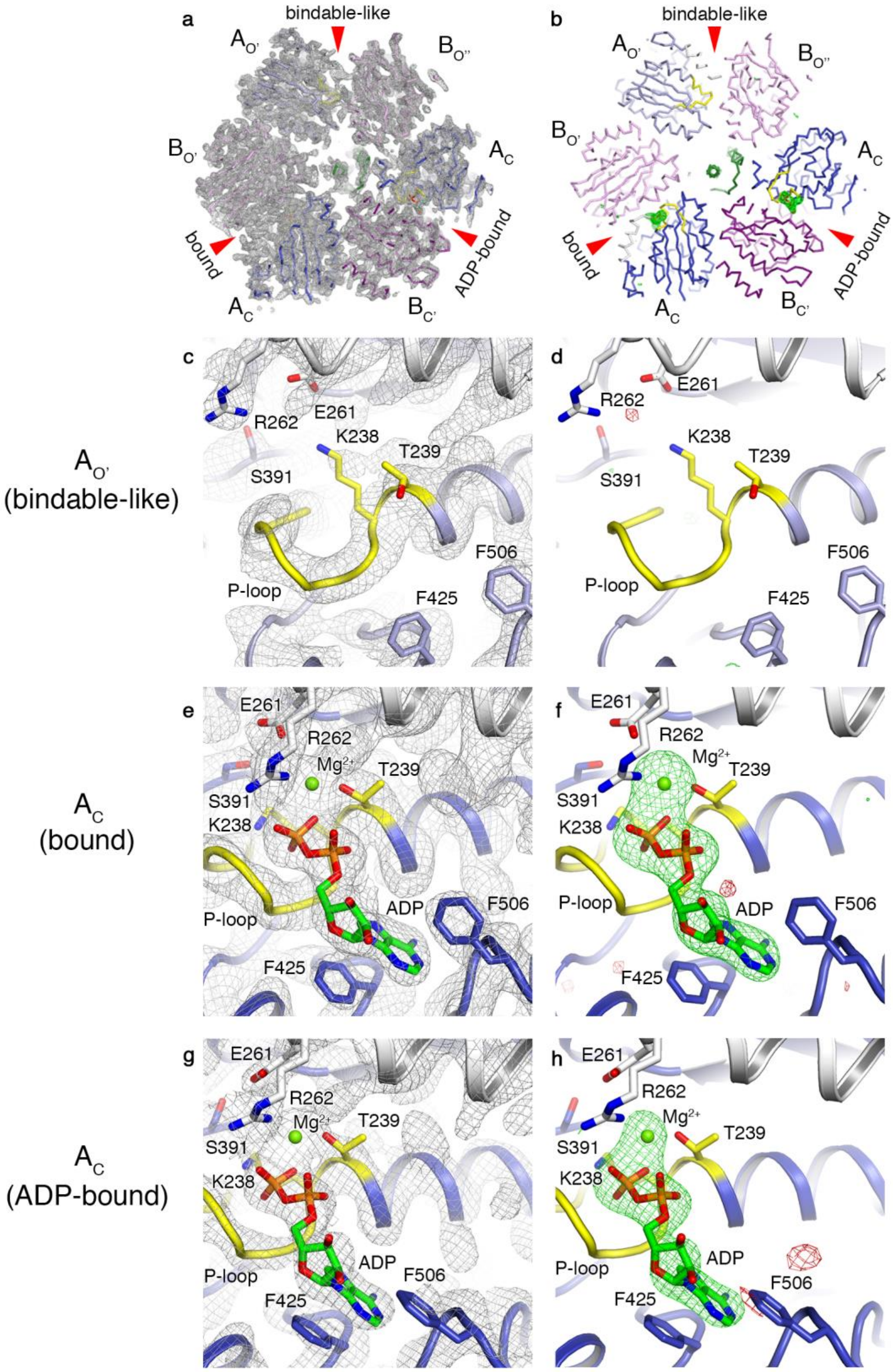

Supplementary Figure 3

Electron-density maps of the nucleotide-binding sites in the 2 ADP-bound V_1 complex ($2_{ADP}V_1$).

(**a**,**b**) Top views of $2_{ADP}V_1$ from the cytoplasmic side at the level of the nucleotide-binding sites. (**c**–**h**) Nucleotide-binding sites of $A_{O'}$ (**c**,**d**), A_C (**e**,**f**), and A_C (**g**,**h**) of $2_{ADP}V_1$ viewed as in the left panel of Figure 4**a**. Residues involved in nucleotide binding are shown in a stick representation. Colours correspond to those described in Figure 4. Left panels (**a**,**c**,**e**,**g**): the 2|Fo|-|Fc| maps contoured at 1.0 sigma are shown in grey. Right panels (**b**,**d**,**f**,**h**): the |Fo|-|Fc| maps calculated without ADP:Mg^{2+} at the binding pockets contoured at 4.0 sigma are shown in red (negative) and green (positive).

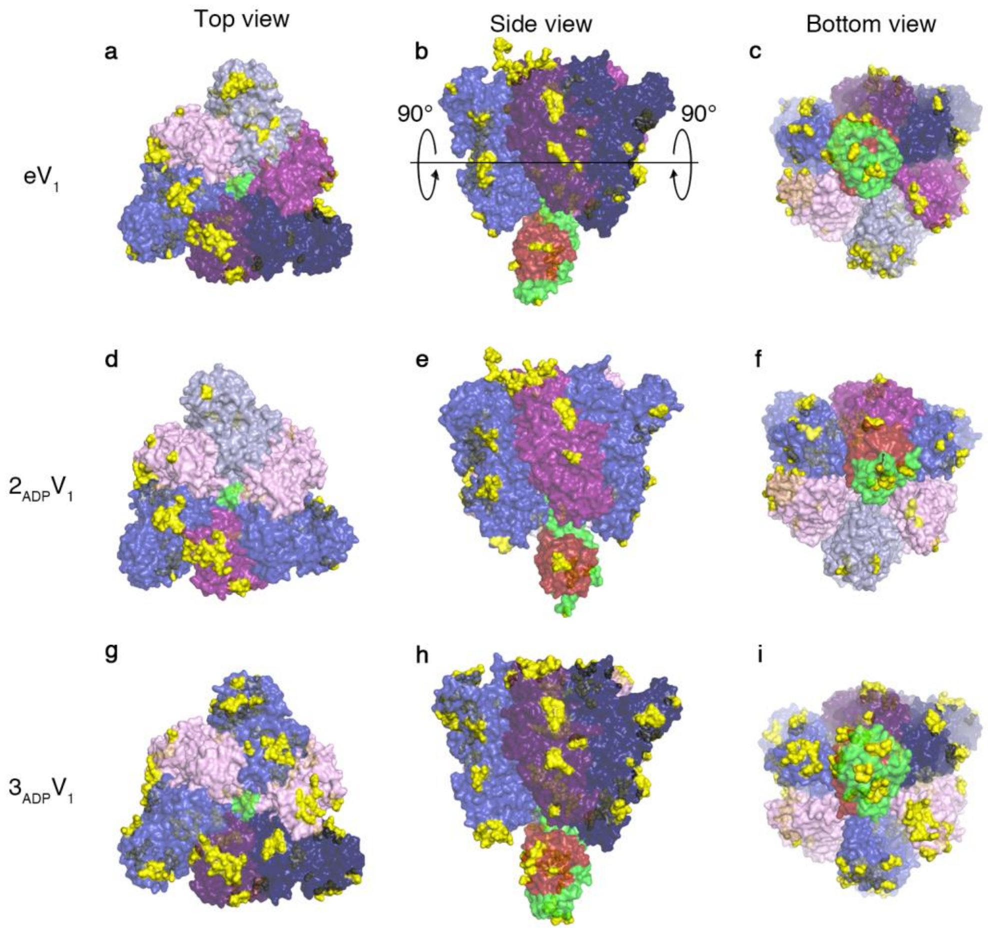

Supplementary Figure 4

Crystal packing contacts of eV_1, $2_{ADP}V_1$ and $3_{ADP}V_1$.

(**a**,**d**,**g**) Top views from the cytoplasmic sides. (**b**,**e**,**h**) Side views. (**c**,**f**,**i**) Bottom views from the periplasmic side. The structures of eV_1 (**a**–**c**), $2_{ADP}V_1$ (**d**–**f**), and $3_{ADP}V_1$ (**g**–**i**) are shown in surface representation. The residues involved in the crystal packing with buried surface area >10 Å^2, as calculated by PDBePISA (http://pdbe.org/pisa/), are shown in yellow.

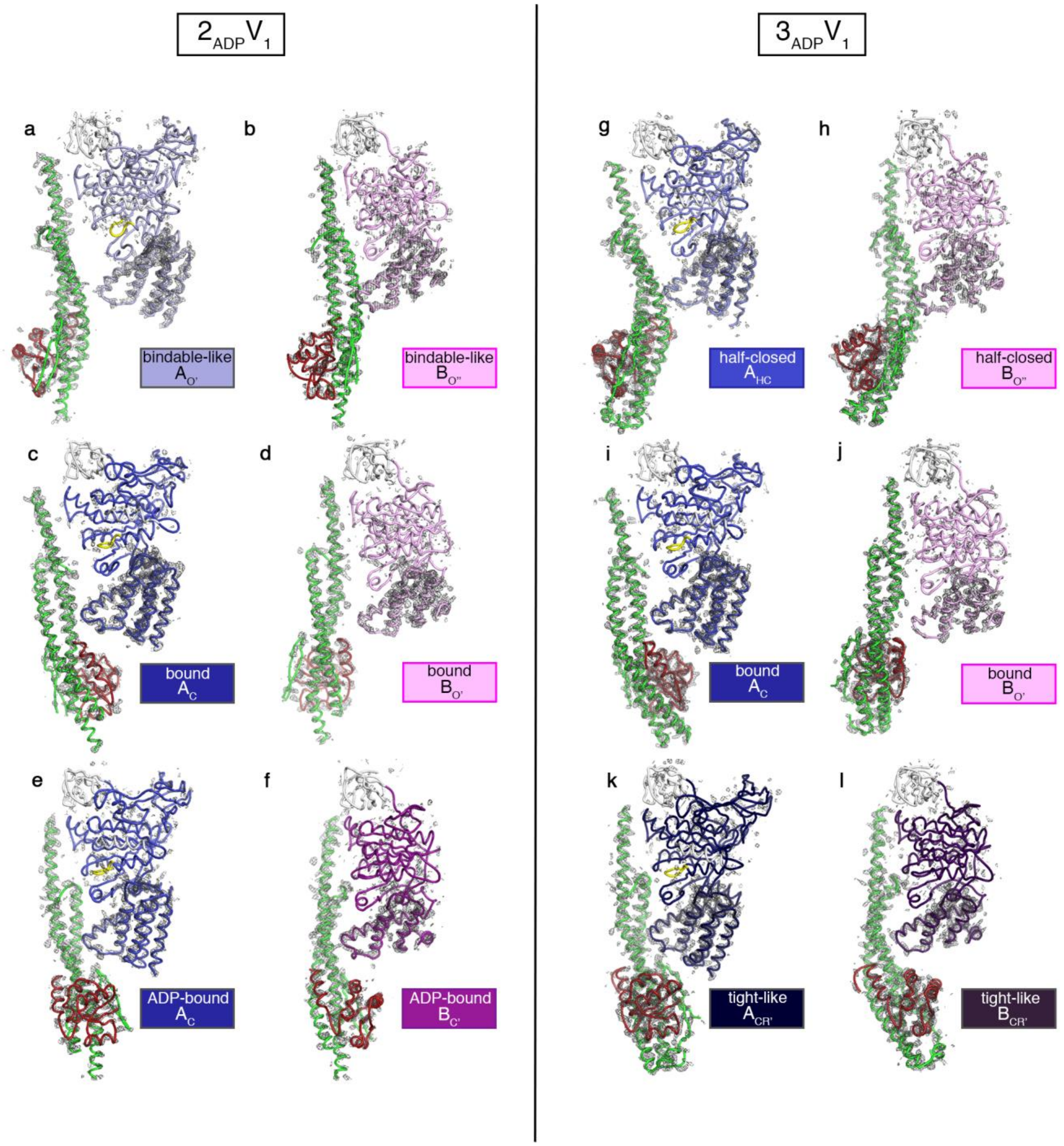

Supplementary Figure 5

Omit electron-density maps of the DF complex and the C-terminal domains of Eh-A and Eh-B of $2_{ADP}V_1$ and $3_{ADP}V_1$.

The structures of $2_{ADP}V_1$ and $3_{ADP}V_1$ that showed conformational changes were validated for possible model bias by making omit maps in the shifted regions, such as the DF complex and the C-terminal domains of the A_3B_3 complex. Eh-A (**a**,**c**,**e**,**g**,**i**,**k**) or Eh-B (**b**,**d**,**f**,**h**,**j**,**l**) with the DF complex of $2_{ADP}V_1$ (**a**–**f**) and $3_{ADP}V_1$ (**g**–**l**) viewed as in Figure 3**c**–**h**. Colours correspond to those described in Figure 3. The |Fo|-|Fc| maps calculated without the DF complex and the C-terminal domains of the A_3B_3 complex contoured at 2.0 sigma are shown in grey.

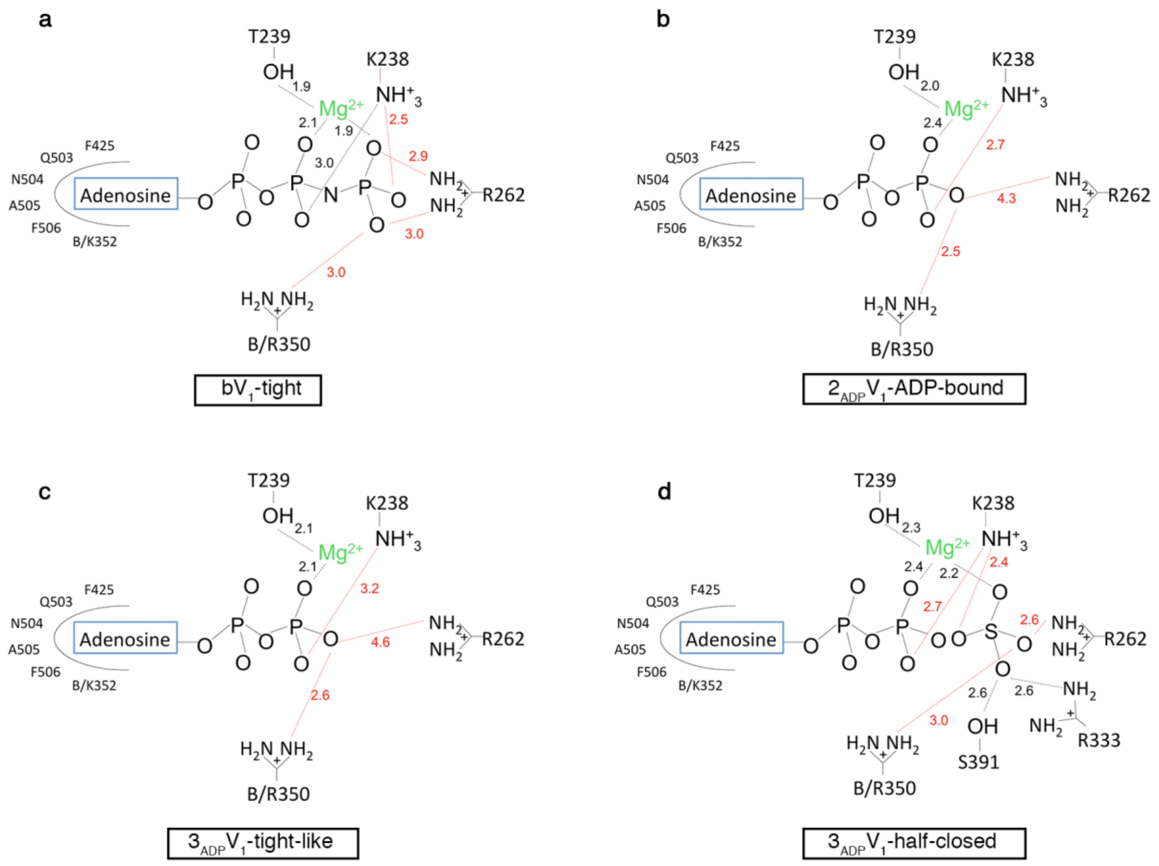

Supplementary Figure 6

Schematic representations of the nucleotide-binding sites of EhV_1.

The distances (Å) between atoms are shown with dotted lines. (**a**) 'Tight' form in bV_1. (**b**) 'ADP-bound' form in $2_{ADP}V_1$. (**c**) 'Tight-like' form in $3_{ADP}V_1$. (**d**) 'Half-closed' form in $3_{ADP}V_1$.

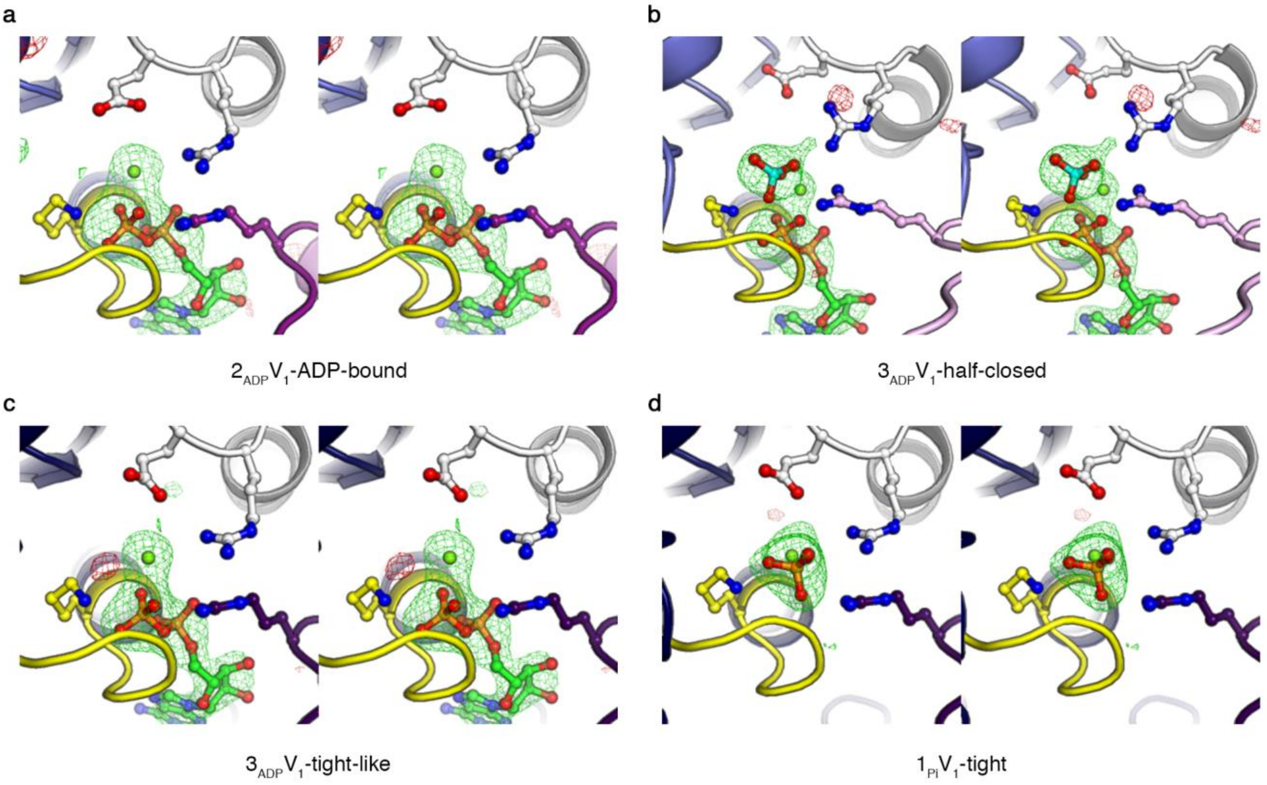

Supplementary Figure 7

Stereo images of nucleotide-binding sites with electron density maps of EhV_1.

(**a**) 'ADP-bound' form of the 2ADP-bound V_1 complex ($2_{ADP}V_1$). (**b**,**c**) 'Half-closed' (**b**) and 'tight-like' (**c**) forms of 3ADP-bound V_1 complex ($3_{ADP}V_1$). (**d**) 'Tight' form of the P_i-bound V_1 complex ($1_{Pi}V_1$). The |Fo|-|Fc| maps calculated without ADP:Mg^{2+} (**a**), ADP:Mg^{2+}-SO_4^{2-} (**b**), ADP:Mg^{2+} (**c**), and P_i:Mg^{2+} (**d**) at the binding pockets contoured at 4.0 sigma are shown in red (negative) and green (positive), respectively. (**a**–**d**) correspond to the right panels of Figure 4**a**, 6**a**, 6**b**, and 7**c**, respectively.

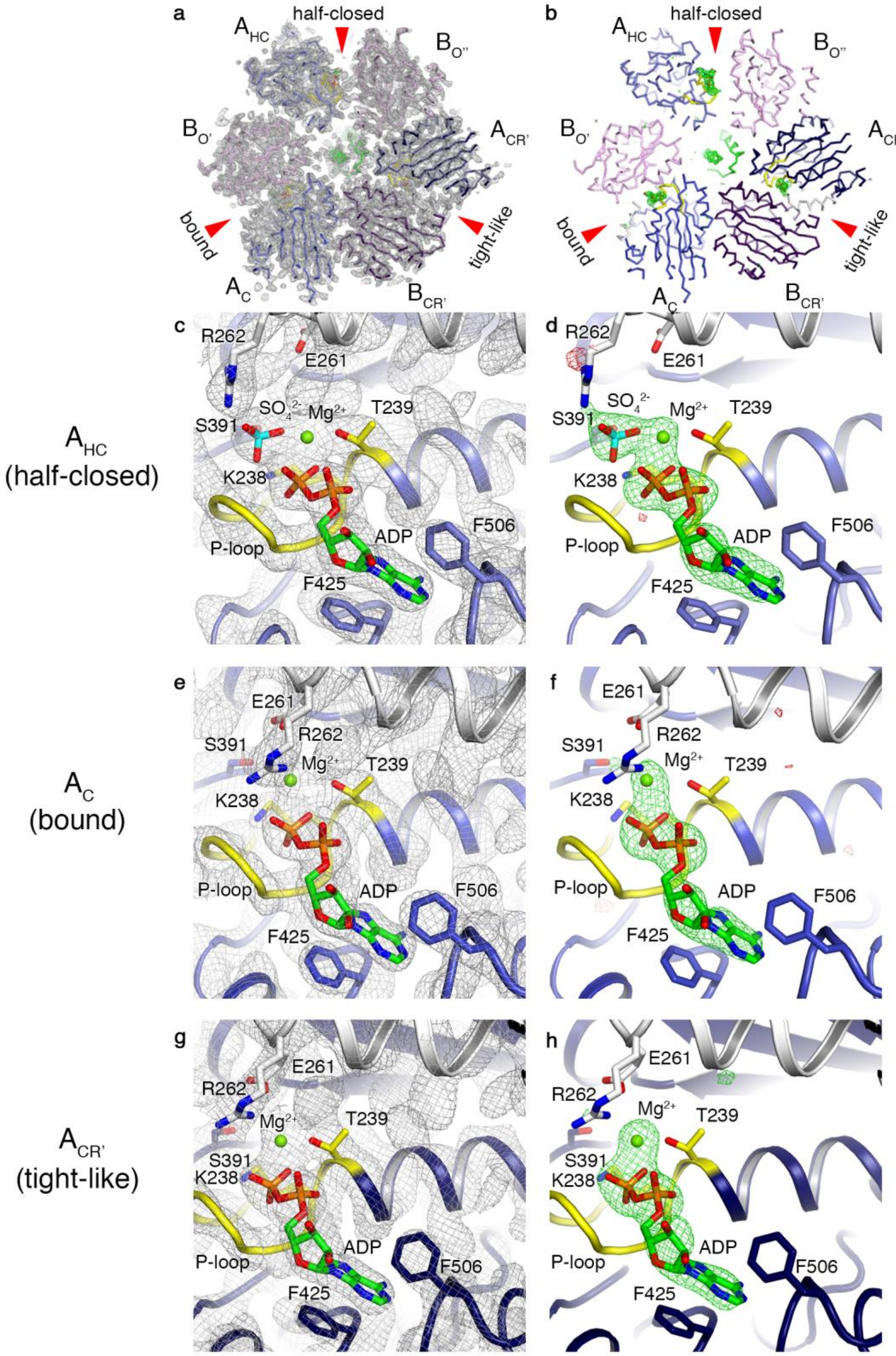

Supplementary Figure 8

Electron-density maps of nucleotide-binding sites in the 3 ADP-bound V_1 complex ($3_{ADP}V_1$).

(**a**,**b**) Top views of $3_{ADP}V_1$ from the cytoplasmic side at the level of the nucleotide-binding sites. (**c**–**h**) Nucleotide-binding sites of A_{HC} (**c**,**d**), A_C (**e**,**f**), and $A_{CR'}$ (**g**,**h**) of $3_{ADP}V_1$ viewed as in the left panel of Figure 4**a**. Residues involved in nucleotide binding are shown in a stick representation. Colours correspond to those described in Figure 4. Left panels (**a**,**c**,**e**,**g**): the 2|Fo|-|Fc| maps contoured at 1.0 sigma are shown in grey. Right panels (**b**,**d**,**f**,**h**): the |Fo|-|Fc| maps calculated without ADP:Mg^{2+} and SO_4^{2-} at the binding pockets contoured at 4.0 sigma are shown in red (negative) and green (positive).

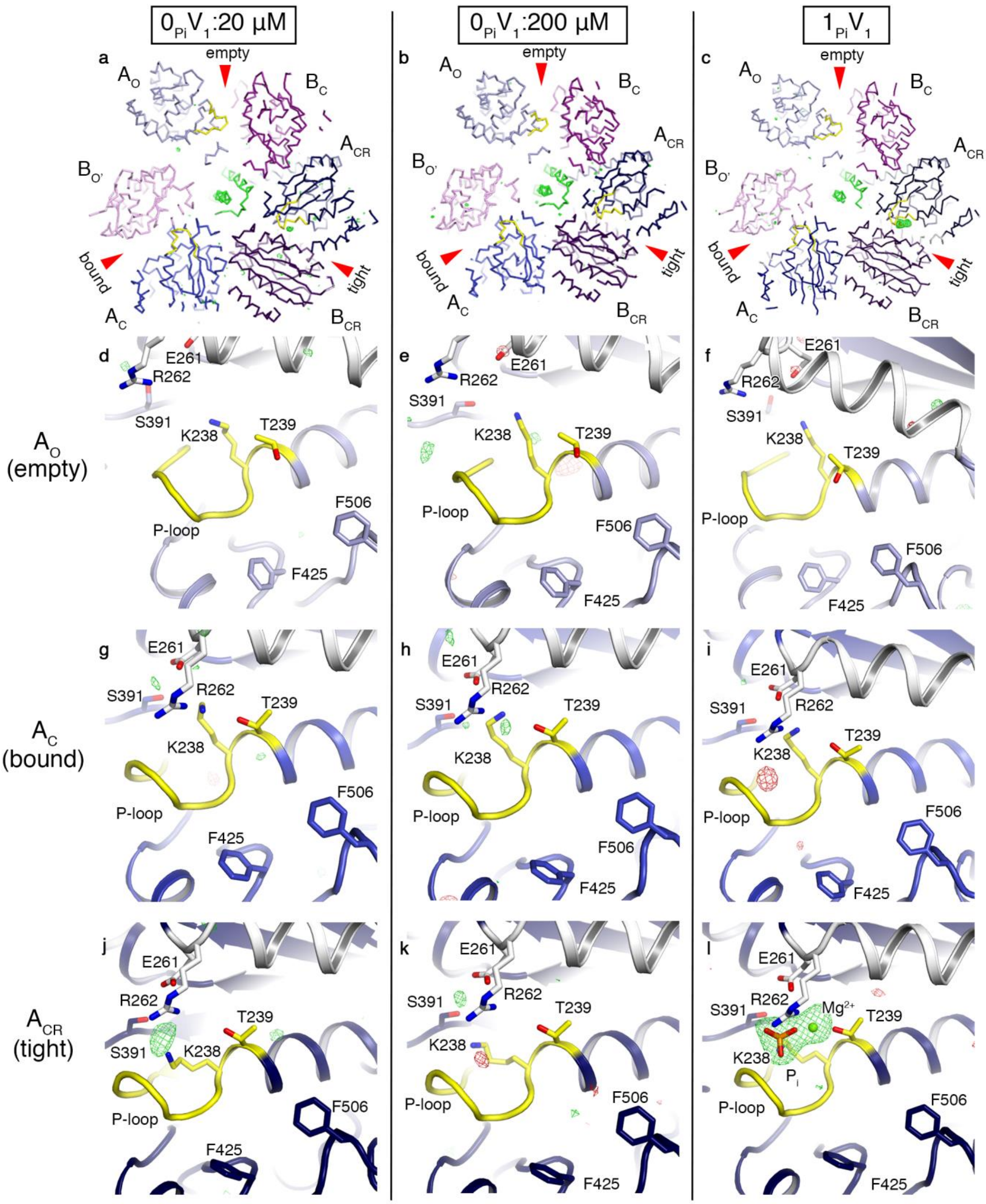

Supplementary Figure 9

Electron-density maps of nucleotide-binding sites in the P_i-bound V_1 complexes.

(**a**,**d**,**g**,**j**; left panels), V_1 complex soaked with 20 μM P_i ($0_{Pi}V_1$:20 μM). (**b**,**e**,**h**,**k**; middle panels), V_1 complex soaked with 200 μM P_i ($0_{Pi}V_1$:200 μM). (**c**,**f**,**i**,**l**; right panels), V_1 complex soaked with 2 mM P_i ($1_{Pi}V_1$). (**a**–**c**) Top views from the cytoplasmic side at the level of the nucleotide-binding sites. (**d**–**l**) Nucleotide-binding sites of A_O (**d**–**f**), A_C (**g**–**i**), and A_{CR} (**j**–**l**) of these V_1 complexes viewed as described in the left panel of Figure 4**a**. The bound molecules (P_i:Mg^{2+}) and residues involved in nucleotide binding are shown in a stick representation. Colours correspond to those described in Figure 4. The |Fo|-|Fc| maps calculated without P_i:Mg^{2+} at the binding pockets contoured at 4.0 sigma are shown in red (negative) and green (positive).

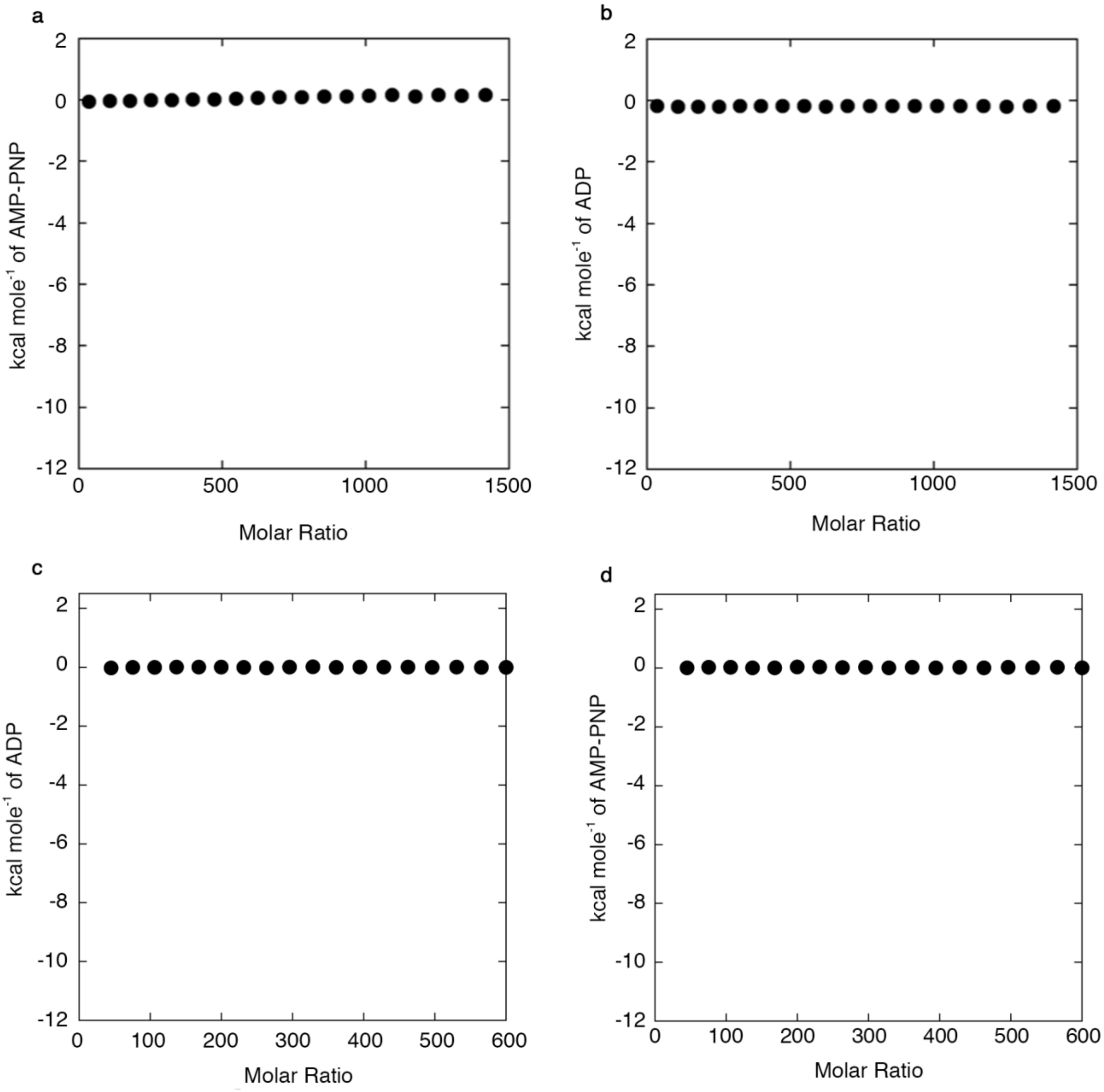

Supplementary Figure 10
Differential binding isotherms of EhV_1 by isothermal titration calorimetry upon addition of high concentrations of nucleotides.
ITC experiments were performed at 25 °C. The integrated heat values from raw heats were plotted against the molar ratio of nucleotide (AMP-PNP or ADP) to EhV_1 after subtraction of the nucleotide dilution heat values from the corresponding heat values for the EhV_1-nucleotide titration. (**a**–**d**) show additional binding isotherms titrated with 15 mM AMP-PNP (**a**,**c**) and ADP (**b**,**d**) to the titrated EhV_1 samples after ITC experiments shown in (**a**–**d**) of Figure 8, respectively.

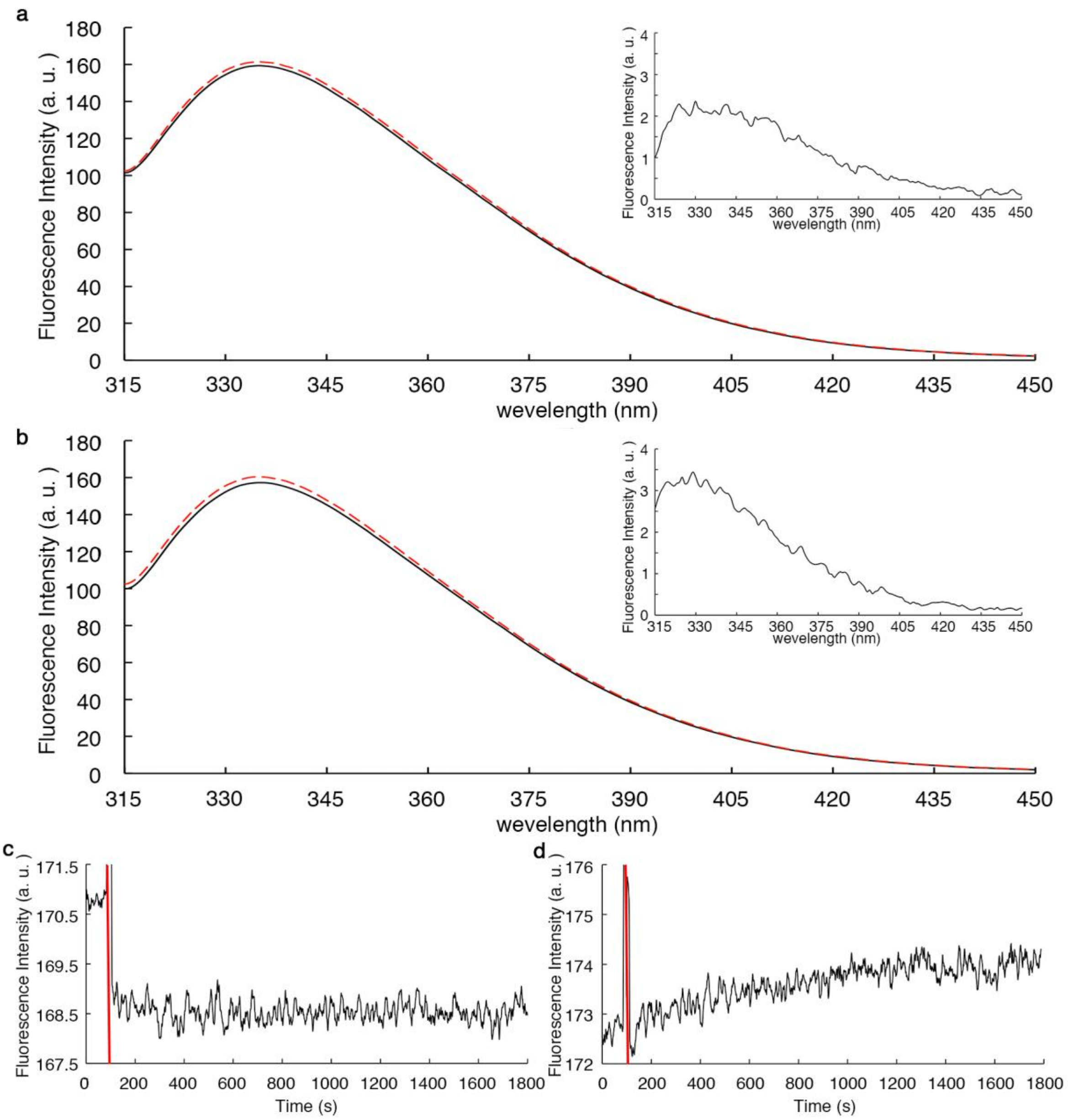

Supplementary Figure 11

The fluorescence measurement of EhV_1.

(**a,b**) Emission spectra of intrinsic tryptophan fluorescence of 100 nM EhV_1 with or without 500 nM nucleotide (**a**: AMP-PNP, **b**: ADP) are shown as a red dashed line and black solid line, respectively. Inset: The difference in fluorescence spectra between nucleotide-free EhV_1 and EhV_1 bound AMP-PNP (**a**) or ADP (**b**). (**c,d**) Time courses of the fluorescence intensity measured at 335 nm. (**c**) 2 mM AMP-PNP was added to ADP-bound EhV_1 at 100 s (red line). The fluorescence intensity decreased to a level very similar to that of AMP-PNP-bound EhV_1, which corresponds to Figure 8**f**, lane 1. Note that Figure 8**f** shows fluorescence intensity between nucleotide-free and nucleotide-bound EhV_1. (**d**) 2 mM ADP was added to AMP-PNP-bound EhV_1 at 100 s (red line), which corresponds to Figure 8**f**, lane 2.

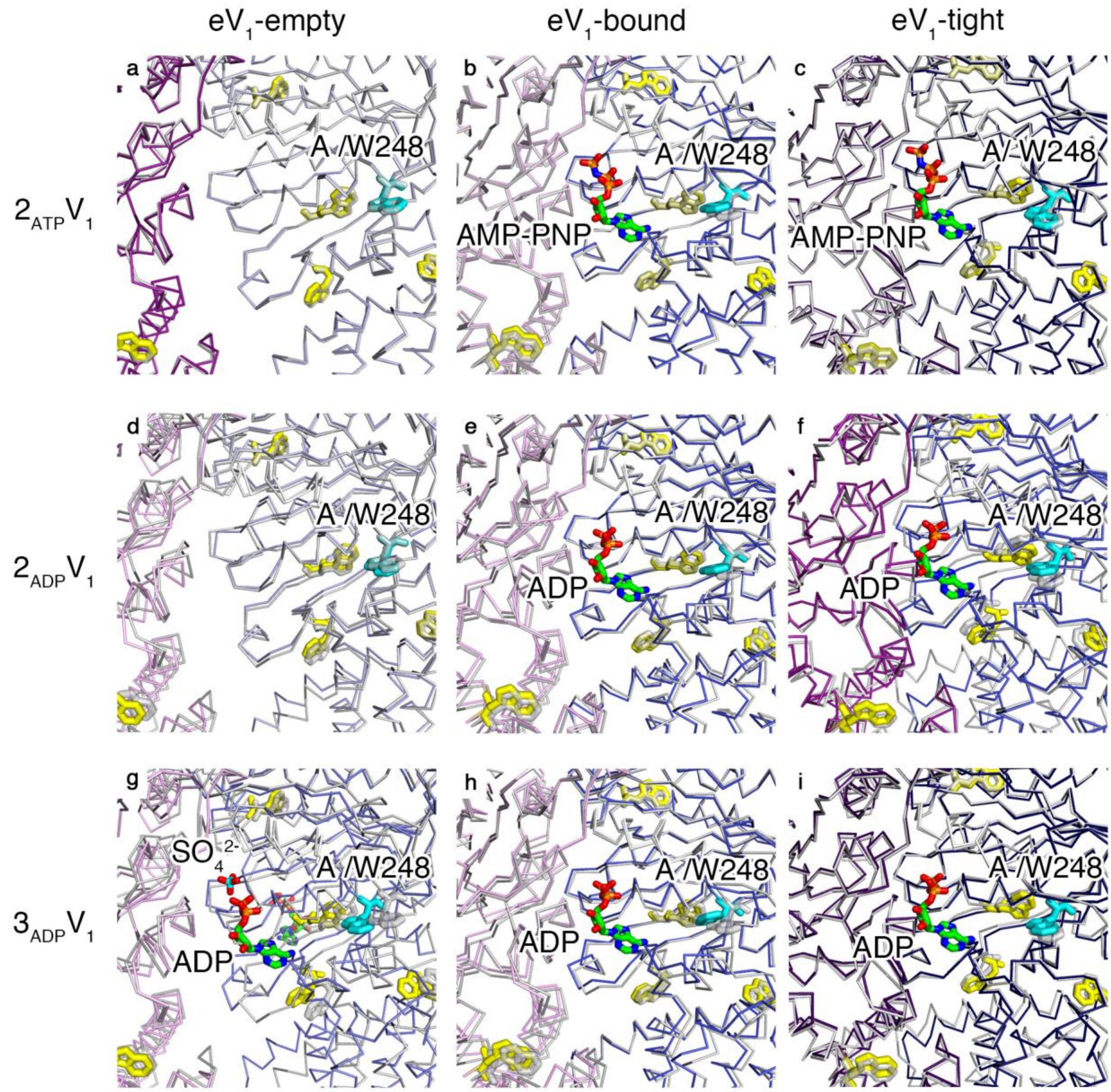

Supplementary Figure 12

Comparison of the conformations of tryptophan residues around nucleotide binding sites of $2_{ATP}V_1$, $2_{ADP}V_1$, and $3_{ADP}V_1$.

The viewing position and colours of these nucleotide-binding sites correspond to the left panel of Figure 4**a**. Trp248 (light blue), other tryptophan residues (yellow), AMP-PNP and ADP are shown as stick representations. (**a**–**c**) $2_{ATP}V_1$ (colour) is superimposed onto the corresponding subunit (grey) of eV_1 using all atoms. (**d**–**f**) $2_{ADP}V_1$ (colour) is superimposed onto the corresponding subunit (grey) of eV_1 using all atoms. (**g**–**i**) $3_{ADP}V_1$ (colour) is superimposed onto the corresponding subunit (grey) of eV_1 using all atoms.

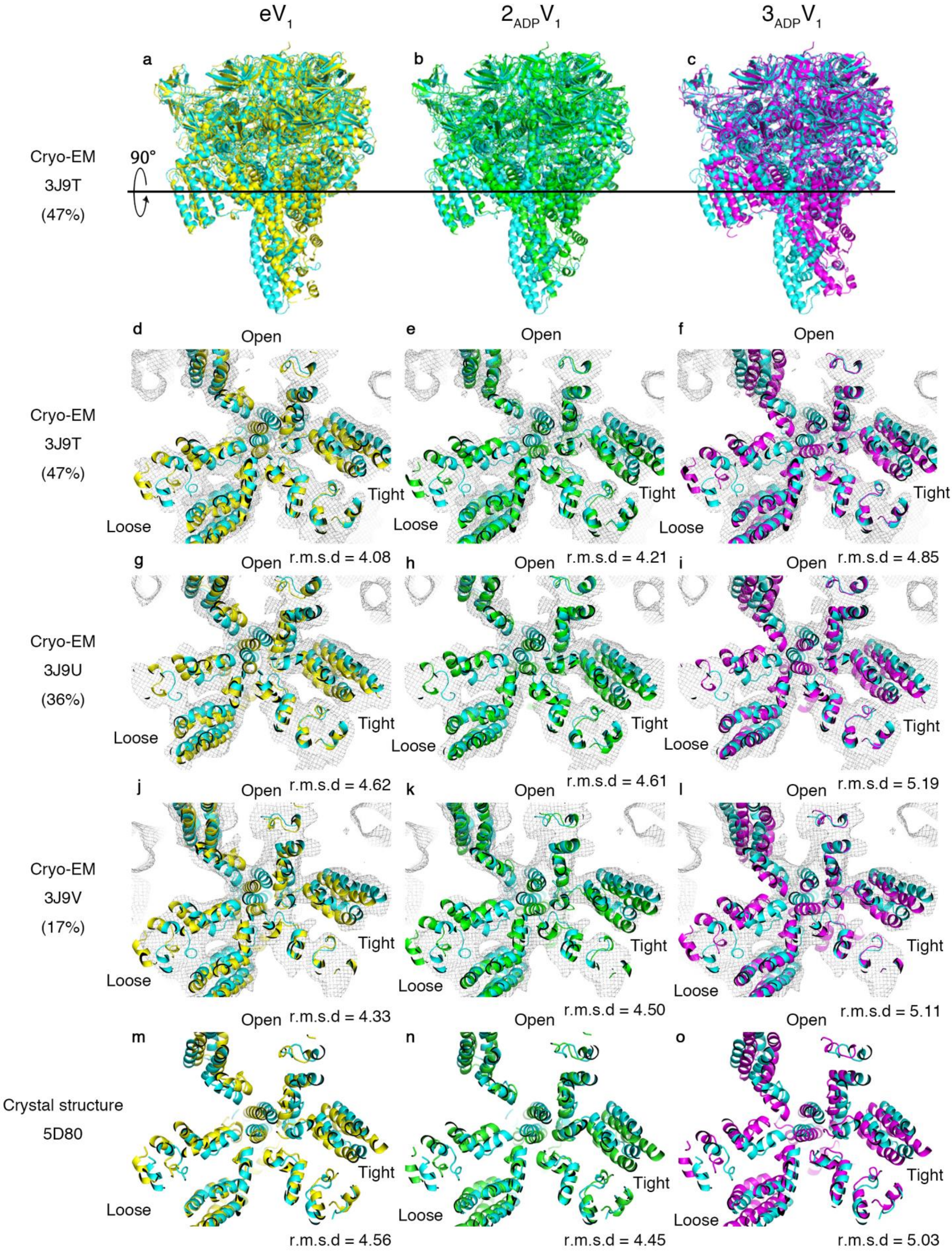

Supplementary Figure 13
Comparison of the structures of *S. cerevisiae* and *E. hirae* V_1-ATPases.

The *S. cerevisiae* V-ATPase models (PDB ID: 3J9T, 3J9U, 3J9V, 5D80)[48, 49], eV_1, $2_{ADP}V_1$, and $3_{ADP}V_1$ are shown in cyan, yellow, green, and magenta, respectively. (**a**–**c**) Side views of the *S. cerevisiae* V-ATPase model (3J9T) obtained by electron cryo-microscopy[48], which are superimposed using Cα atoms of eV_1 (**a**), $2_{ADP}V_1$ (**b**), and $3_{ADP}V_1$ (**c**). (**d**–**l**) Top views of the *S. cerevisiae* V-ATPase model by electron cryo-microscopy (3J9T; **d**–**f**, 3J9U; **g**-**i**, 3J9V; **j**-**l**) from the cytoplasmic side at the level of C-terminal domains, which are superimposed using Cα atoms of eV_1 (**d**,**g**,**j**), $2_{ADP}V_1$ (**e**,**h**,**k**), and $3_{ADP}V_1$ (**f**,**i**,**l**) and fitted to electron microscopy density maps (EMD-6284; **d**–**f**, EMD-6285; **g**–**i**, EMD-6286; **j**–**l**). (**m**–**o**) Top views of the *S. cerevisiae* V_1-ATPase crystal structure (PDB ID: 5D80)[49] from the cytoplasmic side at the level of C-terminal domains, which are superimposed using Cα atoms of eV_1 (**m**), $2_{ADP}V_1$ (**n**), and $3_{ADP}V_1$ (**o**). Root mean square deviation (r.m.s.d) values were calculated using Cα atoms.

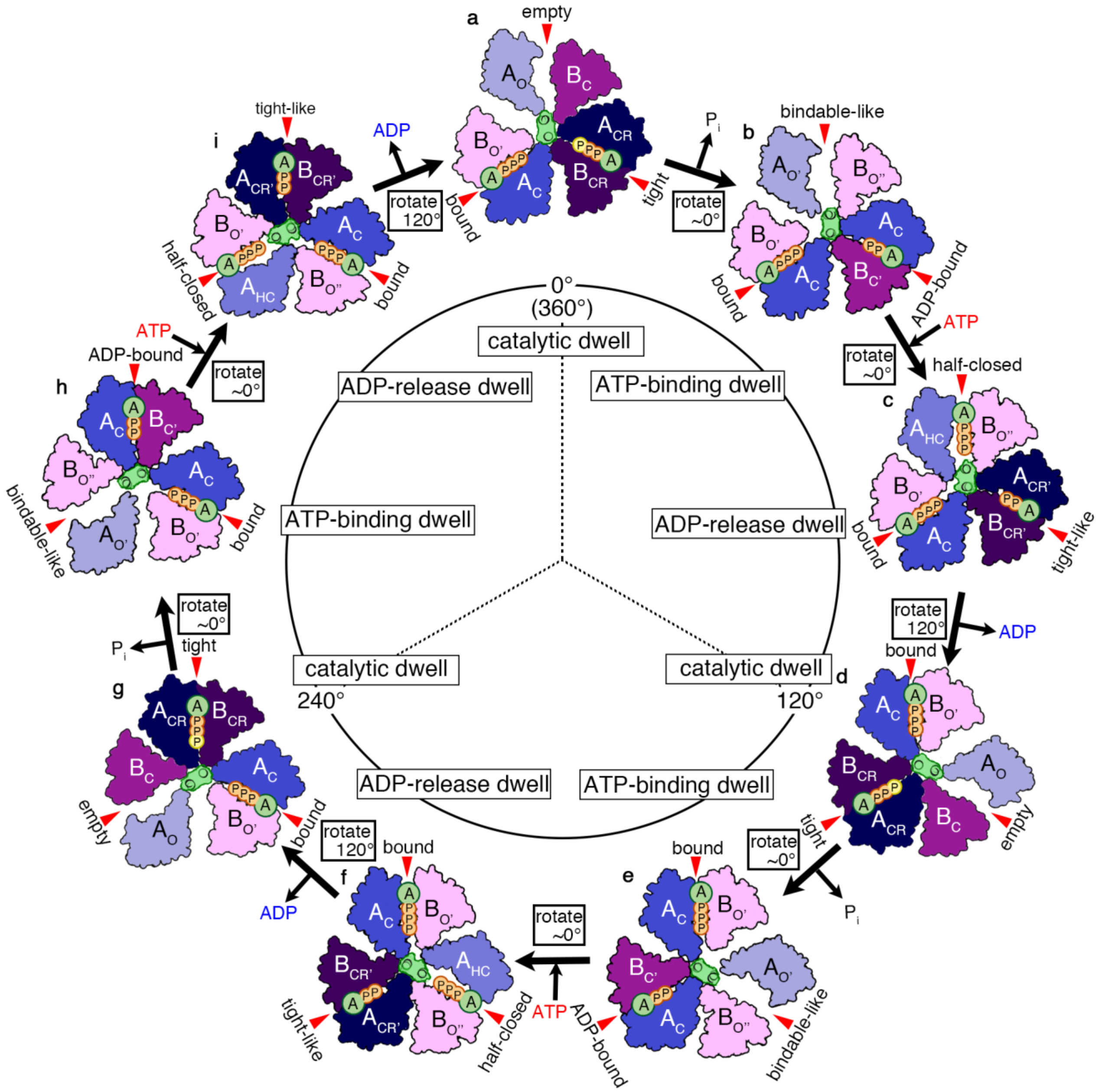

Supplementary Figure 14

A molecular mechanism model for 360° rotation of the V_1 complex starting from the catalytic dwell state.

The structure models are based on the crystal structures of $2_{ATP}V_1$ (**a**,**d**,**g**), $2_{ADP}V_1$ (**b**,**e**,**h**), and $3_{ADP}V_1$ (**c**,**f**,**i**) determined in this study. C-terminal domain views from the cytoplasmic side are shown. ATP with a yellow 'P' represents an ATP molecule that is committed to hydrolysis, as in Figure 9. ATP binds to the 'bindable-like' form at 0° (**b**). It is then hydrolysed to ADP and P_i at 240° (**g**). The P_i molecule with lower affinity than ADP is released at the same angle, and the conformation changes from 'tight' to 'ADP-bound' (**h**). The rest of the ADP molecule is also released at the same angle (240°) induced by ATP binding (**i**).

Supplementary Table 1

		ATPase activity (μmol P_i min^{-1} mg^{-1})	Ratio (%)
Colourimetric method			
control		6.1 ± 0.08	100
AMP-PNP	20 μM	4.3 ± 0.1	70
	200 μM	1.6 ± 0.03	26
	2 mM	-0.40 ± 0.02	~0
ADP	20 μM	6.0 ± 0.5	99
	200 μM	5.2 ± 0.03	85
	2 mM	0.51 ± 0.2	8
ATP-regeneration method			
control		7.8 ± 0.1	100
P_i	200 μM	8.4 ± 0.09	108
	2 mM	8.4 ± 0.1	108
	20 mM	7.9 ± 0.07	101

ATPase activity of the purified V_1-ATPase in the presence of AMP-PNP or ADP was measured by the colourimetric method using molybdic acid. The reaction was initiated by the addition of 1 mM ATP, after a 10 min pre-incubation with various concentrations of AMP-PNP or ADP, and terminated by the addition of 10% sodium dodecyl sulphate. The initial rate of the ATPase reaction at 23 °C was determined within 4 min. ATPase activities of the purified V_1-ATPase in the presence of various concentrations of sodium phosphate (P_i) were measured using an ATP regenerating system. ATP hydrolysis rates at 23 °C were determined in terms of the rate of NADH oxidation, which was measured as a decrease in absorbance of 340 nm. All data represent means ± standard estimated errors (SEM) of three independent experiments. The 100% value of the ratio corresponds to the averaged ATPase activity of EhV_1 in the absence of the inhibitors.

Supplementary Table 2

structure	pair	$2_{ADP}V_1$ bindable-like $(A_{O'}B_{O''})$	$2_{ADP}V_1$ bound $(A_CB_{O'})$	$2_{ADP}V_1$ ADP-bound $(A_CB_{C'})$	$3_{ADP}V_1$ half-closed $(A_{HC}B_{O''})$	$3_{ADP}V_1$ bound $(A_CB_{O'})$	$3_{ADP}V_1$ tight-like $(A_{CR'}B_{CR'})$	$2_{ATP}V_1$ empty (A_OB_C)	$2_{ATP}V_1$ bound $(A_CB_{O'})$	$2_{ATP}V_1$ tight $(A_{CR}B_{CR})$	$1_{Pi}V_1$ empty (A_OB_C)	$1_{Pi}V_1$ bound $(A_CB_{O'})$	$1_{Pi}V_1$ tight $(A_{CR}B_{CR})$
eA_3B_3	empty (A_OB_C)	1.09	3.09	2.36	1.29	3.28	2.65	0.64	3.08	2.75	0.62	3.12	2.73
	bindable $(A_{O'}B_O)$	0.94	2.44	2.72	1.63	2.62	3.30	1.22	2.57	3.28	1.14	2.76	3.30
	bound $(A_CB_{O'})$	2.73	0.59	1.35	1.99	0.73	1.33	3.25	0.80	1.21	3.24	0.71	1.22
bA_3B_3	empty (A_OB_C)	0.95	3.33	2.46	1.31	3.43	2.82	0.59	3.39	2.89	0.61	3.50	2.90
	bound $(A_{C1}B_{O'1})$	2.48	0.60	1.04	2.18	0.59	1.20	2.80	0.63	1.18	2.75	0.72	1.14
	bound $(A_{C2}B_{O'2})$	2.64	0.61	1.01	2.38	0.56	1.12	2.83	0.59	1.10	2.77	0.74	1.10
eV_1	empty (A_OB_C)	1.26	3.45	2.76	1.44	3.37	3.07	0.33	3.29	2.98	0.45	3.50	3.16
	bound $(A_CB_{O'})$	2.97	0.48	1.32	2.32	0.55	1.20	3.15	0.34	1.02	3.08	0.28	0.95
	tight $(A_{CR}B_{CR})$	3.24	0.87	0.68	2.66	1.13	0.60	2.95	0.87	0.34	2.95	0.82	0.38
bV_1	empty (A_OB_C)	1.24	3.32	2.66	1.58	3.59	2.95	0.29	3.56	3.24	0.41	3.39	3.04
	bound $(A_CB_{O'})$	2.82	0.53	1.21	2.47	0.51	1.19	3.21	0.25	0.94	3.09	0.43	1.00
	tight $(A_{CR}B_{CR})$	3.08	0.90	0.69	2.81	1.14	0.49	2.94	0.92	0.26	2.92	1.07	0.38
$2_{ADP}V_1$	bindable-like $(A_{O'}B_{O''})$	0.00	2.56	2.56	0.98	2.58	2.95	1.08	2.70	3.06	1.09	2.99	3.19
	bound $(A_CB_{O'})$	2.56	0.00	0.99	2.28	0.52	1.02	3.10	0.46	0.87	2.96	0.45	0.90
	ADP-bound $(A_CB_{C'})$	2.56	0.99	0.00	2.40	1.06	0.52	2.58	1.13	0.61	2.58	1.29	0.63
$3_{ADP}V_1$	half-closed $(A_{HC}B_{O''})$	0.98	2.28	2.40	0.00	2.34	2.61	1.35	2.41	2.73	1.38	2.32	2.62
	bound $(A_CB_{O'})$	2.58	0.52	1.06	2.34	0.00	1.18	3.21	0.43	1.09	3.24	0.53	1.12
	tight-like $(A_{CR'}B_{CR'})$	2.95	1.02	0.52	2.61	1.18	0.00	2.85	1.13	0.45	2.85	1.23	0.51
$2_{ATP}V_1$	empty (A_OB_C)	1.08	3.10	2.58	1.35	3.21	2.85	0.00	3.11	2.96	0.31	3.19	2.92
	bound $(A_CB_{O'})$	2.70	0.46	1.13	2.41	0.43	1.13	3.11	0.00	0.88	3.21	0.34	0.94
	tight $(A_{CR}B_{CR})$	3.06	0.87	0.61	2.73	1.09	0.45	2.96	0.88	0.00	3.11	1.09	0.26
$1_{Pi}V_1$	empty (A_OB_C)	1.09	2.96	2.58	1.38	3.24	2.85	0.31	3.21	3.11	0.00	3.12	2.93
	bound $(A_CB_{O'})$	2.99	0.45	1.29	2.32	0.53	1.23	3.19	0.34	1.09	3.12	0.00	1.00
	tight $(A_{CR}B_{CR})$	3.19	0.90	0.63	2.62	1.12	0.51	2.92	0.94	0.26	2.93	1.00	0.00

Root mean square deviation (r.m.s.d) values in superimpositions for each AB pair in the crystal structures of the A_3B_3 and V_1 complexes. The values were calculated using Cα atoms.
